# The “Silent Reserves” of the Patriarchal Chinese Welfare System: Women as “Hidden” Contributors to Chinese Social Policy

**DOI:** 10.3390/ijerph17155267

**Published:** 2020-07-22

**Authors:** Yingqi Wang, Tao Liu

**Affiliations:** 1 Center for Social Security Studies, Wuhan University, Wuhan 430072, China; yingqi.wang@stud.uni-due.de; 2 School of Public Affairs, Zhejiang University, Hangzhou 310058, China; 3 Institute of East Asian Studies, University Duisburg-Essen, 47057 Duisburg, Germany

**Keywords:** welfare systems, “silent reserves”, gendering, old-age pension, long-term care

## Abstract

Scholars of social inequality in China have commonly concentrated on strata-related social welfare systems that divide the population into urban and rural dwellers, and additionally, into different welfare classes such as civil servants, employees, and migrant workers. Following Esping-Andersen, Siaroff, Sainsbury, and others, this paper brings the perspective of “gendering welfare” into the study of Chinese social policy. Focusing upon two major social policy branches in China—the old age pension insurance system and care services within the household—it discusses the role of Chinese women in these two fields. Through a gender-sensitive analysis, this paper elaborates the social phenomenon of “silent reserves” (namely, women) within the Chinese welfare regime. While women assume a crucial role in intrafamily care services, constituting the chief contributors of long-term care and childcare, their care contributions at home are not recognized as “social achievements” and are not monetarily compensated by the patriarchal Chinese welfare state. In addition, this paper argues that women are systematically disadvantaged by pension insurance arrangements. Furthermore, the individualization of care services in the intrafamily context weakens the pension entitlements of women, since their unpaid care constrains their ability to maintain full-time jobs in the labor market.

## 1. Introduction

Over the past decades, research on Chinese social policy within international academia has mushroomed, with various studies concentrated on different social policy areas, including several social insurance branches, social assistance, and social welfare [1,2,3,4]. Since the adoption of the reform and opening-up policy, the welfare system in China has been rebuilt and reorganized over the course of market-oriented economic reforms. A comprehensive social insurance program has been established, which includes endowment insurance, medical insurance, work accident insurance, unemployment insurance, and maternity insurance. Further, a social assistance program entitled the Minimum Living Standard Scheme (MLSS) has been set up since the late 1990s, in both urban and rural areas. Besides social insurance and social assistance, social services and social welfare represent other complementary forms of social policy, targeting needy and impoverished people in China. However, the special role of women in the Chinese welfare regime has been largely neglected; social policy studies in China from the gender perspective represent a research lacuna to be filled by theoretically informed and analytical studies. In Chinese academia, very few scholars have grappled systematically with the “gender question” in Chinese social policy and social welfare [5,6]. Internationally, only sporadic discussions have arisen about specific gender-related welfare in China, such as maternity insurance [7] and elderly care [8]. Few of these studies have examined in depth the role of gender in the Chinese welfare regime; most are descriptive and undertheorized, focusing primarily on women’s roles and functions in a single field, such as maternity leave or nursing care. Our research emphasizes a gender-sensitive perspective for the study of Chinese social policy, linking with the general theoretical discussions within international academia about the role of gender in welfare states. The theoretical and conceptual approaches to welfare states and gendering welfare regimes by Esping-Andersen, Siaroff, and Sainsbury constitute the framework of this study, providing a theoretical foundation for looking at the case of China. Since the Chinese welfare state approaches the Bismarck model—a model that is overwhelmingly based on the “social insurance state” (*Sozialversicherungsstaat*)—and China has emulated the German model of social protection since its economic reform, we frequently use Germany as a reference example for the Chinese case in this paper.

We consider two main subfields—the old age pension and care work within households—that are the most relevant against the backdrop of rapid demographic aging [3]. Care work encompasses care services in a broad sense, including elderly care, childcare, and care for disabled family members within households. Further, this essay borrows the term “silent reserves” proposed by the German sociologist Franz-Xaver Kaufmann [9] to highlight the core features of women’s roles in the Chinese welfare state. This approach assumes that the Chinese welfare regime attributes a double role to women. On the one hand, they are the most significant contributors to and suppliers of (unpaid) care work and family chores, especially in the highly feminized sector of elderly care. On the other hand, their work is largely individualized and not yet recognized as a social commitment and contribution. Thus, their significant supporting role and their remarkable volume of work for the Chinese welfare state have been socially “invisiblized”, remaining family affairs. Due to this invisibility, Chinese women have systematically suffered from fragmented working hours and discontinuous working biographies, resulting in precarious payments into their pension insurance accounts and ultimately, a deficiency in the eventual pension. In this sense, Chinese women are the “hidden losers” of the patriarchal Chinese welfare state. Improvement of Chinese women’s social insurance rights—particularly pension entitlements—correlates considerably with the socialization of the feminized sector of family care. The Chinese welfare state must heighten its gender-based sensitivity to remedy the poor social status of Chinese women through establishing a public long-term care protection program.

This study is based on several different sources of data, including statistical data from reports by the Chinese Women’s Association (CAW) and the National Bureau of Statistics of China (NBSC) on women’s social status, as well other statistics from the Sixth Chinese Population Census. One of the primary data sources is the newly published report by the International Labour Organization (ILO), “Care work and care jobs for the future of decent work” (2018) [10], which provides data on education, (paid and unpaid) care work by women, and the uneven involvement of men and women in household chores around the globe, and it includes correspondingly the world regional data and data on China. Through synthesizing these different data sources, we reconstruct the social status and social entitlements of Chinese women, who remain in a precarious situation and who are systematically disadvantaged, regardless of the rhetoric on gender equality formally anchored in the Chinese constitution. A theoretically informed, in-depth analysis of these data demonstrates that China remains a ‘backward’ and masculine welfare regime from the feminist perspective compared to more advanced gendering and feminizing welfare states.

## 2. Theoretical Framework: Bringing Gender back into Welfare State Studies

The progenitor of comparative welfare state studies, Esping-Andersen (1990), touches upon gender perspectives indirectly in his prominent study on the three worlds of welfare capitalism [11]. Different welfare regimes have different institutional arrangements for family policies, which influence women’s social status and social entitlements at least partially. In the liberal welfare regime, market mechanisms are the central institutions regulating and providing benefits for welfare clients, and women’s access to social benefits is predominantly shaped by market-related private and commercial welfare agencies. The inequalities derived from free-market competition have also overshadowed gender inequality in the market economy. Since women are disproportionally employed in the low-income and informal sectors, they are prone to having unduly low pensions and other work-related social insurance benefits. In the conservative-corporatist welfare state, traditional male-centered social policy such as the breadwinner model has endowed women with a passive and dependent role in the welfare state. The conservative welfare state presumes that women should assume the role of homemaker, fulfilling the tasks of care work, and their well-being is highly dependent on the material provisions available through their husbands. The female employment rate in this type of welfare regime is particularly low, women usually do not have independent pension entitlements, and the livelihoods of elderly women mostly rely upon the pension of their husbands, or a small amount of “widow’s pension” [11]. However, gender-related stereotypes and conservatism mirrored the social reality in Continental European countries until the 1970s; since the 1980s, women’s employment rates have continued to rise in conservative welfare states [12]. For instance, the German welfare state has continually increased investments in families and children. A universal child benefit for first-born children was introduced in 1975, and some gender-specific perspectives have been added into the old age pension scheme, such as pension splitting for couples, the recognition of childbearing as qualifying for a pension contribution period, and the introduction of the gender-neutral Riester Pension. Thus, Germany has conceptually transformed from a gender-conservative to a more gender-progressive welfare state [13]. The “conservativeness” of Continental European welfare states has started to fade.

Compared to the first two types of welfare regimes, the Scandinavian social democratic welfare state has fully internalized the avant-garde ideas and values of women’s emancipation, giving political priority to family policies and female employment. Compared to conservative welfare states, social democratic welfare states have comparatively high female employment rates, full-day childcare institutions and agencies are highly developed, and North European welfare states are inclined to combine a high childbirth rate and a high female employment rate [11]. Judged from a gender-sensitive perspective, Scandinavian countries have fulfilled the ideal of gender equality and women’s emancipation, since household tasks and home-based work such as childcare and elderly care are mostly socialized through the welfare state’s generous investment for these traditionally feminized sectors. Due to widely available full-day childcare programs and facilities, Scandinavian women can combine motherhood, childrearing, and employment more easily, and the high female employment rate in Scandinavian welfare states has promoted women’s economic and social independence tremendously (ibid).

Although Esping-Andersen (indirectly) incorporates a gender perspective on social policy, he has been criticized by other scholars for the marginal role of gender in his study [14,15,16]. Siaroff [17] outlines gender differences and integrates gender-related welfare disparities into a comparative study of welfare states. By applying several gender-specific indicators such as the proportion of female college students, female employment rates, the proportion of women in leadership positions, and the share of women in the public sector, he constructs four different types of gender-related welfare states, distinguishing them from the ideal types outlined by Esping-Andersen: the Protestant social democratic welfare state of the Scandinavian region, the Protestant liberal welfare state of the Anglo-Saxon region, the advanced Christian democratic welfare state with Catholic majorities or significant minorities, and the late female mobilization welfare state in Southern Europe. Siaroff argues that Protestantism is an advanced ideational impulse to shape a more gender-equal culture and promote more individualism, self-reliance, and self-fulfillment among women, while Catholicism is conventionally related to conservative values toward employment, family, and gender-related work division, resulting in a more conservative gender culture and public attitudes vis-à-vis women. He concludes that within Scandinavian states (e.g., Norway, Finland, Sweden, and Iceland), all of which are Protestant social democratic welfare states, a progressive and more gender-equal welfare state has emerged, which is characterized by a highly developed public sector including elderly care, full-day childcare facilities and centers (kindergartens) and full-day schools, high female employment rates, and proportionally, the highest share of women’s employment in leadership positions. Mediterranean states such as Italy, Spain, Portugal, and Greece constitute the counter-pole of the advanced Scandinavian welfare states. These countries have comparatively low female employment rates, and their societies strongly rely upon gender-differentiated arrangements and the male breadwinner model. Moreover, women have acquired voting rights only in the very late term and thus are less socially and politically mobilized than women in other types of welfare states. At the ideational level, the Catholic religion and its tradition-oriented gender culture have favored a masculine welfare culture, disfavoring the emergence of a gender-equal feminist or a gender-sensitive welfare state (ibid).

The comparison of welfare states with much more gender sensitivity has demonstrated that different welfare state arrangements, different relationships between state and society, as well as different gender-related beliefs and norms have shaped different types of social rights for women in Western welfare states. In societies in which the market dominates or the traditional beliefs and values (e.g., masculine culture) prevails, women’s social welfare rights have commonly lagged far behind men’s rights. In societies in which social democratic or socialist values have triumphed over other traditional gender-related values, gender-egalitarian social policies will be facilitated. A more gender-based construction and re-construction of welfare states requires a gender-sensitive awakening and an expanding social consciousness and awareness of gender-related social inequality and the willingness of state and society to reduce such inequalities. Such a gender-related reconfiguration of modern society closely dovetails with public sensitivity to and awareness of the gender-based welfare gap.

## 3. The Rudimentary Welfare Regime in China from the Gender Perspective

Conventionally, communist China has been regarded as an advanced gendering regime that seeks to use the communist gender ideology on women’s emancipation to reform and substitute for traditional patriarchal Confucian society [18]. As Mao’s verbal assertion, “Women hold up the half sky”, vividly demonstrates, the new Chinese government after the founding of the People’s Republic endeavored to establish a more egalitarian country from the gender perspective. Even the Chinese constitutions from different episodes guaranteed, and continue to guarantee, full equal rights between men and women in the socialist republic [19]. However, investigating contemporary welfare arrangements in the Chinese welfare state reveals that social inequalities between genders still exist, and the gender welfare gap has perpetuated. Moreover, gender-related social welfare stratification has been further sustained and even promoted in a highly masculine welfare state. There is a remarkable discrepancy between declaratory semantics and the real praxis concerning gender equality and gender welfare.

### 3.1. The “Silent Reserves” and Their Contributions to (Unpaid) Care Work

Traditionally, family chores and care work, including childcare, elderly care, and the care of disabled family members, have been widely regarded as tasks for women. However, in modern welfare states, and especially in Scandinavian countries, female care work and services are considerably socialized and collectivized. The key issue is how and to what extent female care work can be recognized as a social contribution by a state’s public social policy. Another criterium is to see how and to what extent women can be relieved from household activities and to what extent they can participate in the labor market despite their care work within households.

The recently published report by the ILO, “Care work and care jobs for the future of decent work” (2018) [10], has constructed a panorama of the unpaid work of women around the globe. Based on data and the estimation of ILO that include all continents and countries, it identifies a global problem of unpaid work of women. Despite the universality of this problem, gender-based inequality varies among world regions significantly. For instance, men in the Asia-Pacific region perform the lowest proportion of unpaid work within households, amounting to only 1 h and 4 min per day. From the perspective of nation states, men from Pakistan spend the least time in unpaid work at home, amounting to just 28 min; in India, it is 31 min. The mothers of children below 6 years old have been plagued by the most severe “employment penalty”; the employment rate of this group is only about 47.6% worldwide [10]. According to the ILO report, most of the countries in Asia, Africa, and Latin America have not yet launched any kind of public long-term care protection systems, relegating care work to the realm of unpaid work mostly performed by women.

As a member of the Asia-Pacific region, China has experienced similar problems and gender-based inequalities. Applying the data and the estimation provided by the 2018 ILO report, we construct an analytical overview of women’s contributions to care work in China that is informed by the theoretical discussion on gendering welfare states in the previous section.

**Figure 1:**
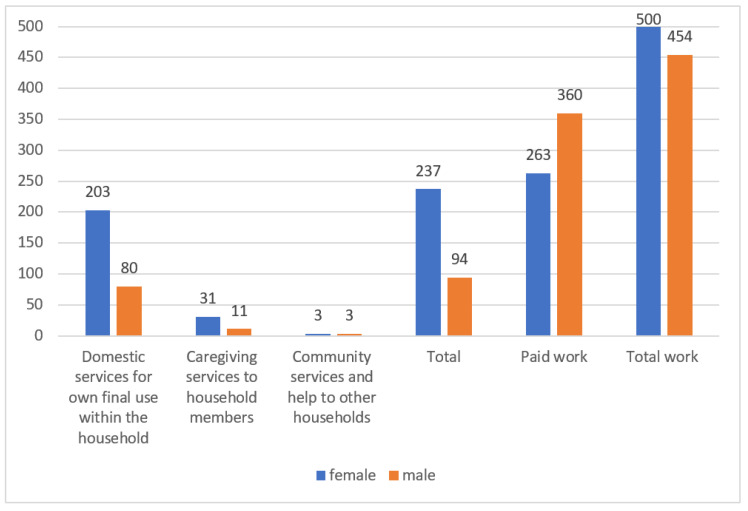
Time spent in the three main categories of unpaid care work and in paid work in China (minutes per day), 2018. Source: Authors’ compilation according to the ILO report (2018): p. 368.

**Figure 2:**
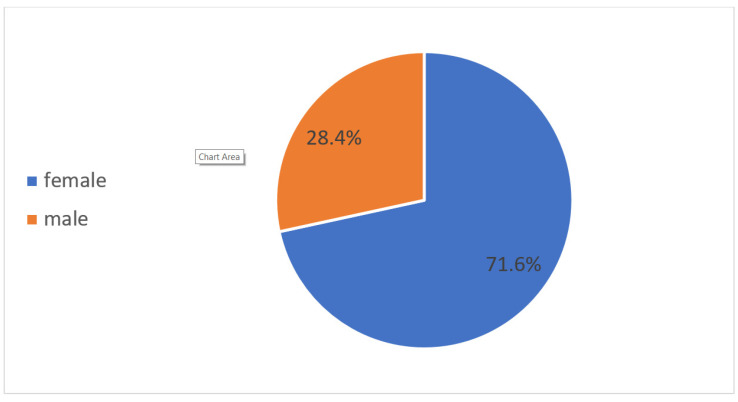
Share of unpaid work in China by sex (percentage), 2018. Source: Authors’ compilation according to the ILO report (2018): p. 368.

**Figure 3:**
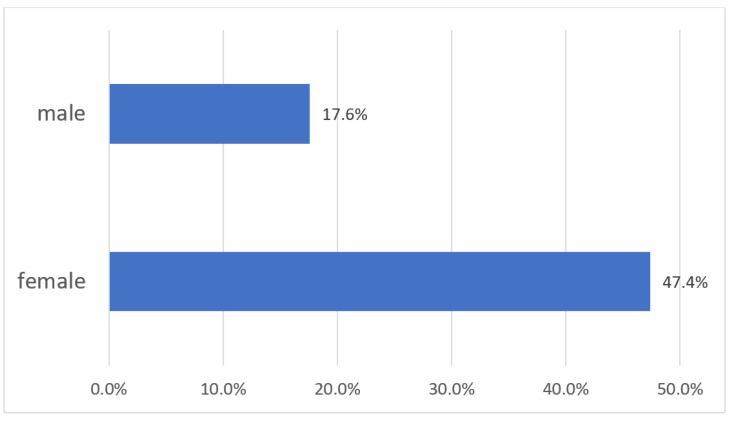
Share of time spent in unpaid work as a proportion of total work time in China by sex (percentage), 2018. Source: Authors’ compilation according to the ILO report (2018): p. 368.

According to the time spent in the three main categories of unpaid home chores—“domestic services for one’s own final use within the household” (such as household accounting and management, food and meal management and preparation, cleaning and maintenance of one’s own dwelling and surroundings, etc.), “caregiving services to household members” (childcare and instruction, care for dependent adults, help for non-dependent adult household and family members, etc.), and “community services and help to other households”—the gender differences in China can be clearly delineated: the time spent by women for domestic services in China is 203 min per day, the time spent by women on caregiving services to household members is 31 min each day, and the time spent on community services is 3 min each day. In total, the time spent by women in these three categories amounts to 237 min per day. By comparison, men spend 80 min each day for domestic services, 11 min for caregiving services at home, and 3 min for community services; the total amounts to 94 min. The average time that Chinese women spend on domestic household work and caregiving services is three times as high as the men’s time investment. The time spent on paid work by women amounts to 263 min per day; for men, it is 360 min. However, if we add the time spent on both paid and unpaid work, women spend in total 500 min per day, which is surprisingly higher than men, who spend in total 454 min (see Figure 1). The women’s share of time spent on unpaid care work amounts to 71.6%, while the men’s share is only 28.4%: more than 43% lower than the women’s contribution (see Figure 2). The share of time spent on unpaid work as a proportion of total work time by women is 47.4%; for men, the percentage is only 17.6 (see Figure 3).

**Figure 4:**
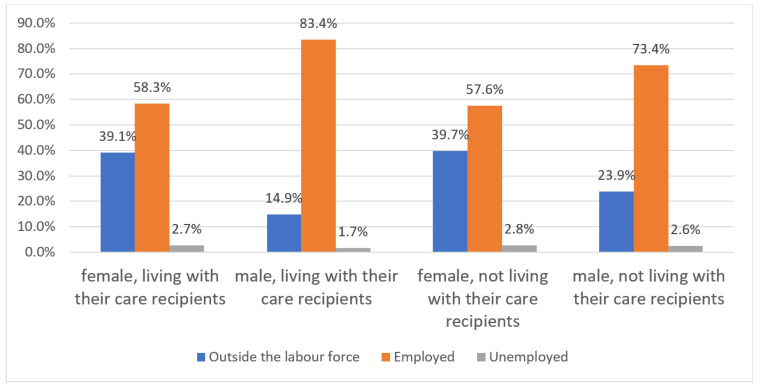
Unpaid care and persons living with and not living with their care recipients in China by sex, 2018. Source: Authors’ compilation according to the ILO report (2018): p. 375.

Among the female unpaid carers who live with their care recipients (such as elderly people who need care and disabled family members), 39.1% are outside of the labor force, 58.3% are employed, and 2.7% are officially registered as “unemployed”. In comparison, among male unpaid care workers living with their care recipients, 14.9% are outside of the labor force, 83.4% are employed, and 1.7% are “unemployed” (see Figure 4). The employment rate of female caregivers living with their care recipients is 25% lower than the male employment rate. In contrast, the proportion of female caregivers living with their care recipients drops many more women from the labor force than men; the difference is nearly three times as high. For the caregivers not living with their care recipients, the gender gap narrows slightly, 39.7% of women and 23.9% of men are outside the labor force, while 57.6% of women and 73.4% of men are employed (Figure 4). The gap in the employment rate narrows to less than 16%; nevertheless, it is remarkable.

**Figure 5:**
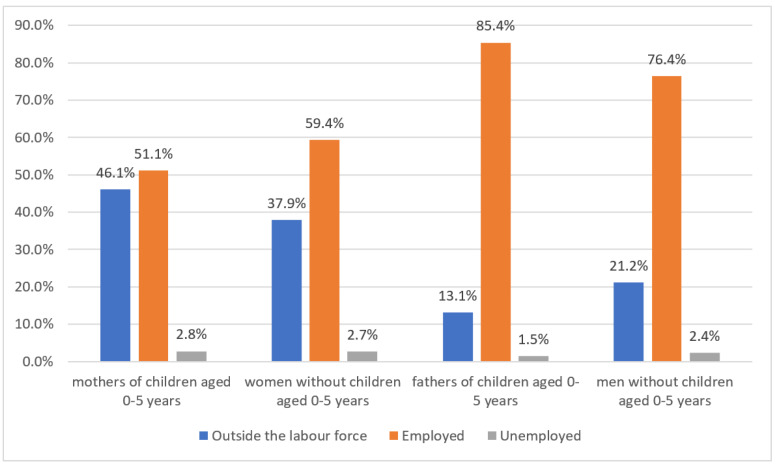
Employment status of mothers and fathers of children aged 0–5 years and women and men without children aged 0–5 years in China by sex, 2018. Source: Authors’ compilation according to the ILO report (2018): p. 392.

More specifically, childcare is a factor that influences women’s employment significantly. Among mothers of children aged 0–5 years, 46.1% are outside of the labor force, 51.1% are employed, and 2.8% are registered as “unemployed”. In comparison, the women without children aged 0–5 years are situated in a more favorable position; 37.9% are outside labor force, 59.4% are employed, and 2.7% are “unemployed”. Compared to fathers of children aged 0–5 years, 13.1% are outside of the labor force, 85.4% are employed, and 1.5% are counted as “unemployed”. Among men without children aged 0–5 years, 21.2% are outside of the labor force, 76.4% are employed, and 2.4% are “unemployed” (see Figure 5). Between these two selected groups—mothers and fathers of children aged 0–5 years—the discrepancy in employment status is most significant: the employment rate of men is 34% higher than for women, while the percentage of residents dropping out of the labor force among women is more than 3.5 times higher than that of men. However, the gender-related gap of employment status between non-mothers and non-fathers of children aged 0–5 years narrows to 17%. In the arena of childcare, the Chinese welfare state again approaches the traditional conservative–corporatist welfare state in Continental Europe. The facilities for childcare are generally insufficient and the care of children is completely individualized; in other words, individual family matters have remained outside the scope of general public welfare since the economic reform in China.

**Figure 6:**
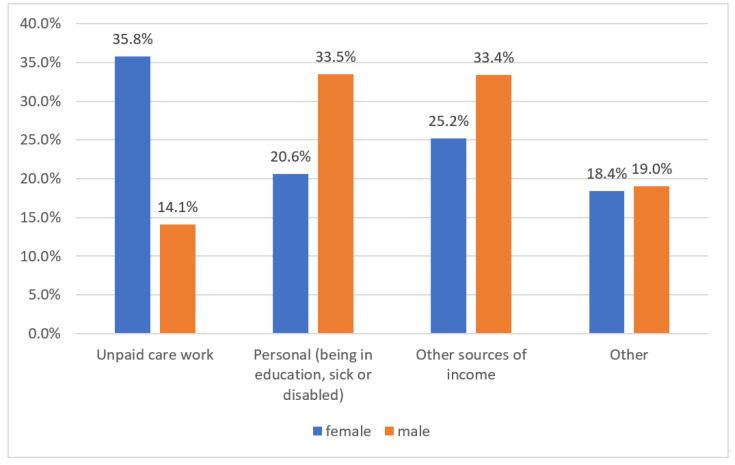
Percentages of inactive persons and the main reasons for being outside the labor force in China by sex, 2018. Source: Authors’ compilation according to the ILO report (2018): p. 386.

**Figure 7:**
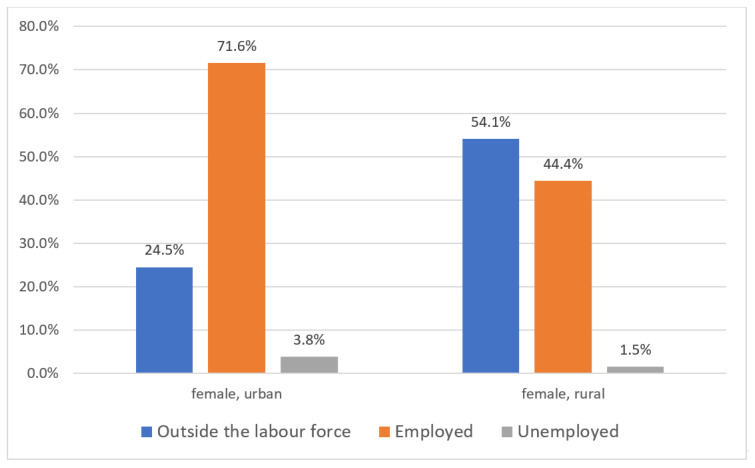
Women as unpaid carers, by places of residence and employment status in China, 2018. Source: Authors’ compilation according to the ILO report (2018): p. 380.

With regard to the reasons for being outside the labor force by sex, the differences are again remarkable: 35.8% of women leave the labor market for unpaid care work, while 20.6% leave for personal reasons such as education, being sick, or being disabled. By comparison, only 14.1% of men drop out of the labor market for unpaid care work, and 33.5% leave because of education, sickness, or disability. The percentage of women outside of the labor force due to unpaid care work is thus 2.5 times higher than men in the same category (see Figure 6). If we consider the urban–rural gap, in urban China, 24.5% of female unpaid carers are outside of the labor force, 71.6% are employed, and 3.8% of them are registered as “unemployed”. In rural areas, 54.1% of female unpaid carers are outside of the labor force, 44.4% are employed, and 1.5% are officially counted as “unemployed”. This urban–rural comparison demonstrates that the percentage of rural female unpaid carers outside of the labor force is twice as high as among women in Chinese cities. The percentage of women who have combined unpaid care and employment in urban areas is 27% higher than in rural areas. Rural women are quantifiably more prone to leaving the labor market due to unpaid care work (see Figure 7). Due to the mass out-migration of the young and middle-age male population from the rural areas, the rural women are much more overloaded by elderly care than the urban women.

The data-based analysis on care and unpaid work by sex reveals that women in China are proportionally much more exposed to the “employment penalty” and risk of unemployment due to unpaid care work for their elderly family members and other kinds of home chores. Since China has still not established formal public long-term care protection programs, as in Scandinavian regions and in Germany, and additionally, due to the complete individualization of childcare within households, Chinese women remain “net silent contributors” to Chinese welfare states. Moreover, millions of Chinese women must leave the labor market because of the heavy burden of care responsibilities within households. With the rapid demographic aging and super-aging and the rising demand of elderly care [20], women’s employment status will deteriorate.

### 3.2. Women’s Pension Entitlements in China

Within a social insurance system, the pension is closely linked to individual employment history and the pension insurance contributions of employees. The benefit level of an old-age pension depends overwhelmingly on the period and amount of these contributions. A gapless and comprehensive employment biography contributes to full pension entitlement and a generous pension; in contrast, a patchy and an incomplete employment biography results in a rudimentary pension entitlement and a fragmentary and precarious pension [21,22]. Since the periods outside the labor force for women are normally much longer than for men, because of the women’s share of care work within the household, women are much more prone to low and marginal pensions and old-age poverty, unless the welfare state takes gender-specific social protection measures to recognize their domestic care work as social work and relieve them at least partially from their home care activities. In traditional Bismarck social insurance programs, most women were precluded from pension entitlements and did not obtain an independent pension, since their sources of income came overwhelmingly from their husbands’ participation in the labor market. In case of the husband’s death, a woman could receive a widow’s pension. However, women’s pension status in Germany has been remarkably improved since the 1970s, with the introduction of a series of gender-related pension arrangements that have elevated the women’s social status in pension entitlement significantly, facilitating a pension-related independence for women [13].

From the perspective of gender-related inclusivity in pension participation, the gender gap in the degree of coverage for Chinese pension schemes has considerably narrowed since the early 2000s. According to the “Report of the Third Survey of Chinese Women’s Status”, conducted by the Chinese Women’s Association (CWA) and the National Bureau of Statistics of China (NBSC) in 2010 [23], 75.9% and 73.3% of urban male and female residents (with non-agricultural Hukou) have participated in old-age pension programs respectively; the gender gap is only 2.6%. For the rural population (with agricultural Hukou), 32.7% and 31.1% of rural male and female residents have participated the old age pension, and the gender gap is only 1.6%. In the “Report of the Second Survey of Chinese Women’s Status”, the data from urban areas showed that in 2000, 65.9% of male residents and 60.5% of female residents were covered by old age pension programs, and the gender gap in coverage was about 5.4% [23]. The gap has narrowed because the Chinese government has introduced non-contributory and tax-financed basic pension programs since 2009 for urban and rural residents who had not been insured by previous social insurance programs [22]. The introduction of a universal pension scheme has considerably improved women’s pension entitlements since they are no longer connected to social insurance contributions; thus, they are also disconnected from employment status in the labor market. Women who are not in the labor market benefit from the non-contributory pension, which is not based on previous work achievements.

However, if judged according to benefit level, the impact of this non-contributory pension on remedying the gender-related pension gap is still limited. Despite the introduction of the non-contributory basic pension for urban and rural residents, its low benefit level has only a marginal effect. Initially, the central government set up a minimum benefit level for the basic pension in the amount of 55 RMB (6.94 €) nationwide, but now, it has been gradually raised to 88 RMB (11.10 €) nationwide, and local governments can top up this minimal pension according to local fiscal resources. In most regions, the benefit level of the basic pension varies mostly from 100 RMB (12.61 €) to 400 RMB (50.44 €). Due to the comparatively low level of the basic pension, it has only limited impact on improving the income and social security of female seniors. The contribution-financed old age pensions still play a significant role in safeguarding the livelihood of senior citizens. In short, the gender gap in pension insurance payments persists, and it is still significant.

According to the calculations by Gao and Pan, the old age pension income of Chinese women is only 56.23% of men’s income [5]; thus, women are much more exposed to social risks and old-age poverty than male pensioners. According to the “Report of the Third Survey of Chinese Women’s Status”, the average labor income of women in urban and rural areas, respectively, is only 67.3% and 56.0% of the average income for men. The percentages of women in the low-income group in urban and rural areas are 59.8% and 65.7% respectively, which are 19.6% and 31.4% higher than male employees. Since the Chinese old-age pension insurance in urban areas consists of two pillars—the social pooling financed by a pay-as-you-go financial model and individual pension accounts financed by mandatory individual pension payments consisting of 8% of personal gross income—the lower income of women is automatically related to lower pension payments into the individual pension accounts. Consequently, due to the gender wage gap, women have lower average pensions than men. Considering that China still adheres to an outdated model of retirement age, 60 years for men and 50–55 years for women (The retirement age for men and women was established after the founding of the People’s Republic of China. At that time, life expectancy in China was comparatively low. Female employees in enterprises retired regularly at 50 years old and female civil servants and female staff in public institutions usually retired at 55 years old. This model of very early retirement for women has persisted to the present day and inevitably, it has a significant impact on the benefit level of women’s pensions.), the early retirement age for women further diminishes the contributions paid into their individual pension accounts. Generally, female pensioners in China receive much lower amounts from the pension insurance system than male pensioners. Since women have a longer average life expectancy than men, the gender-related pension injustice becomes even more salient.

The population censuses in China have also included gender-related data on social security and old-age pension income. According to the Sixth Population Census of China (2010), among the population aged 60 and over nationwide, 28.89% of men’s income came from old-age pension payments, compared to 19.58% for women. In urban areas, the share was 74.21% for men and only 59.07% for women. At the level of townships, the figures were 35.24% and 17.85%, respectively. In rural areas, only 2.09% of women’s income came from old age pension payments; the percentage for rural men was three times higher—7.19% (see Table 1). Due to these comparatively low pension levels, elderly women have become more dependent on other income sources outside the institutional state pension to sustain their livelihoods, and the elderly women in China rely much more upon the supply of family members than elderly men. An independent female pension is still a big social question in the current Chinese social protection.

## 4. Conclusions

This research has revealed a gender-related paradox in the development of welfare in China. As a self-proclaimed “socialist state”, China had borrowed the radical communist gender ideology to reform Chinese society in the 1950s, facilitating women’s emancipation and social participation between the 1950s and the 1970s. However, especially since the reform and opening-up period, gender-related welfare in China no longer adheres to the advanced and progressive script of “women’s emancipation”; in contrast, gender-related rhetoric in the reform era has surprisingly continued to backpedal, hearkening back to the ideals of conservative welfare states (prior to the 1980s). Chinese society is anchored to a conventional model of gender-related “social normality”, which has ascribed certain pre-described and ascriptive roles and tasks to women, such as domestic care work. Based upon our analysis concerning the nexus of unpaid care, employment status, and employment rates for men and women, the concept of women as the “silent reserves” within the welfare state proposed by Kaufmann applies to China. Women’s participation in household chores and care work constitute their most significant contributions to the patriarchal Chinese welfare state; however, these contributions are mainly “invisible” and indiscernible. Thus, women’s care contributions remain “silent”, and to a certain extent, neglected in a male-dominated welfare system. Women’s articulations of their interests and their welfare rights are still too weak to be heard. Women’s capacity of discourse-making in the field of social welfare is still negligible.

The widespread social perception of “social normality” in China, which is based on the ascriptive and prescriptive social roles of women, has neglected a basic social fact—namely, that this type of gender-related “normality” is socially constructed—and it also reflects the differential power relationship between men and women in a patriarchal society. If domestic care activities were not performed by female family members and these household care demands had to be externalized and satisfied through public social agencies, the externalization and socialization of home care would engender an incredible amount of social costs that would have to be financed either by public tax revenues or social insurance contributions. From this perspective, women are significant contributors not only to their families, but also to the entire welfare state through their significant but invisible contributions to the national human capital. If these hidden costs were made socially “visible” and explicit, the society and the state would need to redirect public resources and invest a considerable portion of the country’s GDP into the care sector. The social welfare system and long-term care arrangements in China can only function if women remain willing to be the “silent reserves” of Chinese welfare state. If social semantics and scripts such as those concerning increased women’s participation and integration into the labor market and society undergo a social revival and become prevalent in Chinese society, the conventional model of a patriarchal welfare state would no longer function. The key issue is the explicit recognition of female home and care work as social contributions.

However, gender-correlated sensitivity in the realm of social protection and welfare in Chinese society remains low. Thus far, only a small number of scholars has integrated a gender perspective into research on social insurance systems such as old-age pension insurance. Within international academia, gender-related issues such as long-term care and elderly care have been increasingly investigated; however, the gender power gap and the patriarchal features of care systems have been largely neglected. Gender-related inequality and asymmetry have been rarely incorporated into the general study of care and welfare in China [25,26,27,28]. Nor have any powerful advocacy coalitions attempted to put gender-related pension schemes and semantics onto the current political agenda. From a gender perspective, the welfare state in China is, surprisingly, furthest from the progressive gendering welfare state (exemplified by the so-called Protestant social democratic welfare states in the Scandinavian region). Indeed, the social welfare configuration in China proves far removed from the socialist gender ideology to liberate suppressed women. The lack of gender sensitivity in the Chinese welfare state makes it akin to the “late female mobilization welfare state” in the Mediterranean region [17] and the conservative welfare state [11]. In fact, the Chinese welfare state shares rather the commonality with the early form of the conservative welfare state (pre-1980s). The basic system architecture of the Chinese social insurance system (especially old-age pension insurance and the current pilot projects on the long-term care insurance system) still adheres to the traditional Bismarck model of social insurance, relying overwhelmingly on the male breadwinner model that inevitably divides society into two gender-based classes. The gendering upper class—the male members of society—usually has a gapless and continuous work biography that results in generous pensions, while the gendering underclass—society’s female members—have been severely disadvantaged through the many gaps and loopholes in their work biography created through temporary and permanent unpaid care work within households.

At the ideational level, counterviews against the introduction of cash benefits for family members who care for their parents and relatives (such as the German “Pflegegeld” ( Pflegegelder—in English literally “long-term care allowances”—relate to the cash payments that are transferred to family members who care for the elderly people within the household in Germany.) of the long-term-care insurance system) is still prevalent in Chinese society. A full socialization of home care services for elderly family members and children, as in the progressive Scandinavian welfare state, is still unpopular in the Chinese society. Few academics have advocated for the externalization of costs related to domestic care services, since this narrative does not correspond to the “social normality” of Confucian culture, which relegates care for family members and relatives to women. Mainstream opinions and semantics rather underpin only rudimentary long-term care services directed at the severe problems that cannot be resolved within families. There is little interest in a narrative of women’s emancipation that benefits the re-entry of women into the employment market. Against this backdrop, the recent experimental long-term care insurance pilot projects do not seek to relieve Chinese women from the tasks of unpaid care fundamentally; they rather seek to provide limited external assistance and so-called “short-term breaks” for female care providers within households. Even after the national long-term care insurance system will be introduced in 2020, elderly care will remain a highly feminized sector within families. Female caregivers remain systematically disadvantaged in the social insurance system, since they cannot take part in the social insurance scheme fully and they do not have formal employment status due to their activities invested in domestic care services. The introduction of a universal and non-contributory pension since 2009 is probably the right first step from a gender perspective, since all rural and urban residents can receive a small amount of pension disconnected from their previous achievements and contributions in the labor market. Indirectly, women in China have benefited from this new type of social pension. However, due to the extremely low benefit level, this tax-financed citizen pension has only a limited impact on the improvement of the pension status of Chinese women.

Judged from a more gender-sensitive angle, China remains a “backward” gendering welfare state despite its self-proclaimed gender-equalization narrative derived from the Chinese communist movement since the 1920s. China needs more gender advocacy coalitions and powerful lobby groups to enlighten the public about the problem of gendering welfare and to enhance the sensitivity and awareness of this still marginalized issue. Among existing social and political organizations, the All-China’s Women’s Federation (*zhonghua quanguo funü lianhehui*) and its local associations and branch organs are the core institutions that represent women in China, defending and promoting their interests. Such entities must additionally elevate this issue onto social and political agendas efficiently, as well as convince Chinese policy makers to change women’s social welfare status quo. There is still a long way to go for China to become a gendering welfare state that cares more for the interest of women and changes their positions as the “silent reserves” and “hidden” contributors to the Chinese welfare state.

## Figures and Tables

**Table 1:** Sources of income for persons aged 60 and over in China by sex and by place of residence (percentage), 2010.

Main Sources of Living	National		Cities		Townships		Rural Areas	
	Male	Female	Male	Female	Male	Female	Male	Female
Labor income	36.59	21.92	9.72	3.75	29.23	15.71	50.53	32.14
Old-age pension	28.89	19.58	74.21	59.07	35.24	17.85	7.19	2.09
Payments from the Minimum Living Standard Scheme	4.11	3.69	1.76	2.87	4.1	4.39	5.14	3.85
Property income	0.41	0.44	0.75	0.62	0.58	0.43	0.21	0.16
Supply by other family members	28.24	52.59	12.13	31.95	28.74	59.39	35.13	59.93
Other	1.76	1.90	1.44	1.83	2.12	2.23	0.27	0.03

Source: The Sixth Population Census of the People’s Republic of China by the National Bureaus of Statistics of China [24].
